# Drivers of Farmers’ Intention to Use the Digital Agricultural Management System: Integrating Theory of Planned Behavior and Behavioral Economics

**DOI:** 10.3389/fpsyg.2022.901169

**Published:** 2022-10-21

**Authors:** Sangluo Sun, Xiaowei Wen, Siqiong Jie, Qihua Gao, Ying Zhu, Simei Wen

**Affiliations:** ^1^College of Economy and Trade, Zhongkai University of Agriculture and Engineering, Guangzhou, China; ^2^Research Center for Green Development of Agriculture, South China Agricultural University, Guangzhou, China; ^3^College of Economics and Management, South China Agricultural University, Guangzhou, China; ^4^Sloan School of Management, Massachusetts Institute of Technology, Cambridge, MA, United States

**Keywords:** farmers’ management behavior, digital agricultural management system, adoption intention, behavioral economics, behavioral biases, theory of planned behavior

## Abstract

China’s fishery industry has national and international relevance whose aquaculture production accounts for more than 60 percent of the world’s total aquaculture production. But the average amount of pesticides used per hectare in China is roughly five times of the world average. The abuse of chemical fertilizers and drugs has brought chronic, long-term, and cumulative harm to both human beings and environment. The digital agricultural management system should be adopted to reduce non-negligible environment pollution and the quality and safety risks of aquatic products. So, it is essential to understand the factors that may influence the adopting intention of this digital management approaches. The present study aimed to examine the adopting intention of farmers toward the digital agricultural management system using two theories–the theory of planned behavior (TPB) and the behavioral economics–as the research framework. The population was composed of farmers in the provinces of Guangdong province in south China of whom 219 farmers were sampled with stratified random sampling technique. Structural equation modeling was used to analyze the data, and it was revealed that this research framework could potentially predict intention. And we observed that the two biased belief of availability bias and loss aversion bias can be the main predictive influence factors of responsible behaviors in adopting the digital agriculture management system, which highlights the importance of framing recommendations in terms of losses rather than gain may be more effective to increase farmers’ intention to adopt the digital system on their farms.

## Highlights

– Examining the farmers’ adopting intention toward the digital agricultural management system by combining the theory of planned behavior (TPB) and the behavioral economics.
– Two biased beliefs of availability bias and loss aversion bias can be the main predictive influence factors of responsible behaviors.
– It is important to frame recommendations in terms of losses rather than gain may be more effective to increase farmers’ intention to adopt the digital system.

## Introduction

For over 60 years, global apparent food fish consumption has increased at a rate significantly above that of world population growth. Per capita fish consumption in 2018 currently stands at 20.5 kg, underlining the critical role of aquatic food in global food nutrition and safety (FAO, 2020). China is one of the world’s largest country of consumption and cultivation of aquatic products, so aquaculture development in China plays a vital role in the global food supply. According to the Data from China MOA, in 2018, China’s aquatic production reached 64.802 million tons, including 50.5 million tons of aquaculture production, accounting for more than 60 percent of the world’s total aquaculture production. In 2019, the total aquaculture production of China reached 50,797,700 tons, accounting for 78.4 percent of the country’s total aquatic production (FAO, 2020; Ministry of Agriculture and rural Affairs of the People’s Republic of China, 2020).

However, the average amount of pesticides used per hectare in China (13.1 kg per hectare) is roughly five times of the world average (2.6 kg per hectare) (FAOSTAT, 2017). According to the 1,031 food unqualified data released by the Chinese State Administration for Market Regulation in 2020, there were 3,799 unqualified batches of edible agricultural products, among which 1,148 batches of aquatic products were unqualified, accounting for 30.22%, which means that, although China ranks among the world’s largest aquatic producers, there is much room for improvement. In China, small-scale aquaculture production is predominant, resulted in low productivity and profitability. With the continuously increasing density of aquaculture, various aquatic diseases outbreak more frequently, which leads to the phenomenon of overuse and misuse of drugs. So, the recurrent problems of food contamination in China are due to illegal chemical additives purposely with the pursuit of profit instead of unintended infectious agents or environmental toxins (The Lancet, 2012; Wen and Liu, 2012). Drug residues in aquatic food caused by drug abuse will bring non-negligible, chronic, long-term, and cumulative harm to both human beings and environment, which emphasize the need for adopting more efficient mange-control approaches.

The digital agricultural management system establishes quality safety risk assessment and an early warning mechanism based on advanced technology, such as the Internet of things, big data, cloud computing, chain blocks, AI and 5G, which is one of the most significant ongoing transformation processes in global modern agriculture and food chains (El Bilali and Allahyari, 2018; Groher et al., 2020). With the different types of sensors, the data from the breeding process and the water and soil environment can be collected easily. The technologies of 5G and Internet of Things are used to store and calculate data with 5G video monitoring, micro-environment monitoring, physiological changes monitoring to realize the real-time monitoring, and agricultural big data management. In addition, with the intelligent real-time monitoring of temperature, humidity and water quality, the digital management system can also realize intelligent early warning, quality control, and other functions to improve the intelligent automatic control of the whole breeding process. Not only can it enhance the effectiveness of supervision and decrease management costs, but also it can assist to improve the traceability chain to force farmers to take responsibility and inform farmers about the correct and scientific aquaculture method, increasing resource productivity, reducing the quality and safety risks, improving environment pollution, and contributing to agro-food sustainability transition (El Bilali and Allahyari, 2018; Weersink et al., 2018; Zhao et al., 2019). However, small-scale individual aquiculture is the dominant aquaculture model in China at present, which leads to a low degree of aquiculture scale, low productivity, and a low profit rate. Therefore, in order to pursue profits and meet their livelihood needs, farmers not only constantly increase the density of aquaculture but also use drugs excessively or improperly, leading to the deterioration of water ecological environment and frequent occurrence of diseases. Based on the field survey, the use of digital system by aquatic farmers in the country is extremely low, while farmers still take a consistent traditional strategy in agriculture production. To implement and adopt the digital agricultural management system at the farm level generally requires a behavioral change from the farmers. However, behavioral change is difficult to induce and sustain, even it is affordable and practical (Panter-Brick et al., 2006; Ellis-Iversen et al., 2010). Behavioral interventions are more effective when they are aimed at important antecedents of behavior and at removing barriers for change (Steg and Vlek, 2009). This emphasizes the need to get a more in-depth understanding of the factors that may affect farmers’ adoption intention of digital agricultural management system.

Therefore, this research aimed to examine the factors that influence farmers’ adoption intentions by combining two well-known behavioral theory, the theory of planned behavior (TPB), and behavioral economics. The TPB asserts that behavioral achievement relies on both the motivation to engage in a behavior and the ability to engage, and individual’s intention is determined by 3 central socio-psychological constructs: attitude, perceived behavioral control, and subjective norms (Ajzen, 1991). In the field of agriculture, TPB was used to find out the adoption of conservation practices (Borges and Lansink, 2016; Jiang et al., 2018), farmers’ business decisions (Stojcheska et al., 2016; Daxini et al., 2018; Nguyen and Drakou, 2021), and the intention and behavior of production and breeding (Sun et al., 2017; Wang et al., 2018, 2021; Grilli and Notaro, 2019; Rezaei et al., 2019; Lima et al., 2020; Ataei et al., 2021).

However, it assumes that all behavior is rational, which requires a high level of cognitive effort and fails to consider irrational determinants of human behavior, especially behavior of farmers who have low-level cognitive capacity (Conner and Sparks, 2005; Mingolla et al., 2021). Many pieces of evidence show that individuals are “predictably irrational,” making biased decisions that defy traditional economic theory, ultimately not in their best interests (Ariely, 2010). Heuristics and cognitive biases will also affect farmers’ intention (Lam et al., 2017). In addition, behavioral economics challenges the fundamental assumption that humans behave as fully informed and rational actors, and human decision is generally influenced by behavioral biases (Simon, 1972; Frederiks et al., 2015; DellaValle, 2019; Jenssen et al., 2019; Carminati, 2020). It has recently been suggested to be a promising theory to better understand farmers’ adoption intention and behaviors (Huijps et al., 2010; OECD, 2012; Wolf, 2017; Mingolla et al., 2019).

**FIGURE 1:**
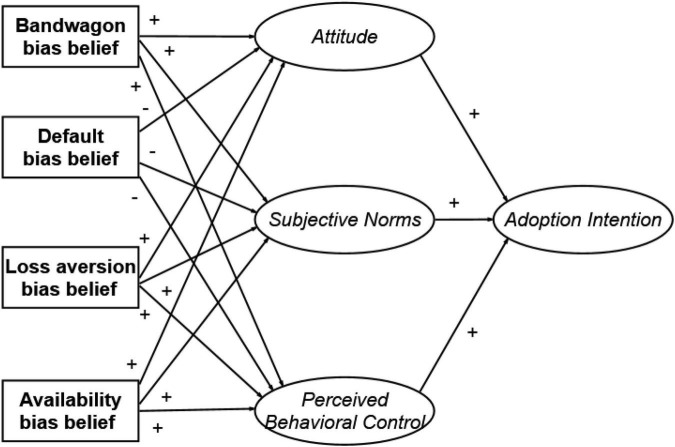
A conceptual model. The determinants of the TPB are indicated in font italic; the biased beliefs are indicated in font bold.

Many related literature and studies showed that TPB and behavioral economics have long been used to explore individuals’ behaviors and intentions, but no research has been conducted with respect to farmers’ intention to adopt the digital agricultural management system according to the TPB and behavioral economics in the form of an integrative or separate model. So, this study may fill another gap of past worldwide studies on the digital agriculture. According to the analysis, Figure 1 presents the theoretical research framework and the hypothesized relationships of this study, relating the four biased beliefs to the three TPB determinants. By cognitive processing of this study, the general goal is to determine the factors influencing farmers’ willingness to adopt the digital agricultural management system among Chinese farmers. For achieving this general goal, the following specific aims are pursued:

- Exploring how farmers’ behavioral biases influence their behavioral intention toward the digital system.
- Providing more empirical evidence and realistic information on Chinese farmers’ intention to adopt the digital system with the application of TPB and behavioral economics.
- Providing coherent planning and policymaking in this regard.

## Materials and Methods

### Sample Selection and Data Collection

The population of interest consisted of all aquaculture farmers in Guangdong Province. Guangdong occupies the aquaculture leadership position nationally, with 8.76 million tons aquaculture production in 2020, accounting for 13.37% of the total and ranking first in China, considered to be an important aquaculture region of China.

With MOA’s help, we were able to access the contact list of aquaculture farmers in Guangdong for our in-person survey. Those aquaculture farmers were divided into five homogeneous groups according to their counties, and the sample size of each group was determined according to the proportion of population of each county. The stratified proportional sampling method was used for sampling randomly to ensure the representability of each aquaculture farmer sample. After sampling, based on guidelines of “TPB Questionnaire Construction” (Ajzen, 2002; Mingolla et al., 2019) and the methods of *The Power of Survey Design* (Iarossi, 2006) to ensure the accuracy of responses and the participation of respondents, two structured questionnaires were designed for aquaculture farmers. Each interview lasted around 35 min. In total, 219 samples from 27 villages in 5 counties were then selected by random sampling with proportional allocation during November 2020 to January 2021 (a response rate of 87.6%).

### Measurement Instrument

The main instrument in this research was a questionnaire, which measured TPB-factors and a biased belief with existing and validated scales adopted from the “TPB Questionnaire Construction” guide (Ajzen, 2002; Mingolla et al., 2019). In order to assure whether operational definitions of the constructs were correctly presented and whether the items could subjectively/theoretically cover the constructs, face and content validity were examined (Gravetter and Forzano, 2012), with an expert group, including professors of agricultural sciences, environment, and psychology, to evaluate the indicators. And we have done the corrections and confirmed the questionnaire on the basis of the experts’ views to enhance the readability, completeness, relevance, and clarity of the questions. As Table 2 shows, the values of all constructs were either close to or above 0.70, showing adequate reliability of the questionnaire (Nunnally, 1978).

**TABLE 1:** Descriptive statistics of respondents’ characteristics.

Item	Mean	S.E.	Minimum	Maximum	N
Gander (1 = male, 2 = female)	1.37	0.485	1	2	219
Age	37.68	10.607	17	67	219
Work years	7.15	6.285	1	34	219
Family size	4.94	1.748	1	11	219
Know other apps (1 = yes, 2 = no)	1.32	0.469	1	2	219
Total sample	–	–	–	–	219

**TABLE 2:** Descriptive statistics of key variable indicators.

Variable	Mean	SD
**Attitude to the digital system (ATT) α = 0.799**		
Good	3.12	1.23
Helpful	3.31	1.06
Improve efficiency	3.44	1.04
**Subjective norms (SN) α = 0.748**		
The opinion of others about the digital system is important to me	3.33	0.99
The opinion of experts about the digital system is important to me	3.36	1.01
The advice and information of important referents about the digital system is important to me	3.25	0.98
**Perceived behavioral control (PBC) α = 0.837**		
The decision of accepting the digital system is under my control	3.34	0.94
I can learn to use the digital system easily	3.31	0.96
I can use the digital system whenever I want	3.38	0.94
**Availability bias belief (ava) α = 0.786**		
You ever caused a loss due to non-use of medication or under-use of medication	3.37	0.96
You ever caused a breeding disease of not taking drugs/under-use of drugs	3.26	0.92
**Loss aversion bias belief (los) α = 0.761**		
The time and effort to use the digital system would be too much for me	3.32	0.89
The cost of using the digital system would be too high for me	3.26	0.97
**Default bias belief (def)**		
I always apply the same treatment	3.3	0.92
**Bandwagon bias belief (ban)**		
Other farmers hold a positive attitude toward the digital system	3.37	0.92
**Adoption intention (ADO) α = 0.752**		
I am willing to change my default way for the digital system	3.36	0.87
I am willing to adopt the digital system	3.36	0.91

*The questionnaire was designed by using a Likert 5-grade scale, and the options were composed of five levels from strongly disagree to strongly agree. Default bias belief and bandwagon bias belief are manifest variables without α.*

The final survey questionnaire was divided into four general parts. The first part included the characteristics of individuals and farms of the farmers (e.g., age, gender, farm size, etc.). The respondents’ age ranged from 17 to 67, with an average age of 37.68, while the average working experience is 7.15 years. The second part included a series of questions designed to measure TPB-factors and consisted of nine items in three subdivisions: attitude (α = 0.799), subjective norm (α = 0.748), perceived behavioral control (α = 0.837), with three items per construct. In the third part, the biased beliefs were measured, consisting of six items. Availability bias (α = 0.786), loss aversion bias (α = 0.761), and bandwagon bias and default bias all showed to be reliable constructs. The respondents were asked to indicate the extent of their agreement or disagreement with statements made to measure the variable (based on Likert scale: from 1, strongly disagree, to 5, strongly agree, reducing statistical problems) (Fornell, 1992). The fourth part measured farmers’ intention to adopt the digital agricultural management system, which is the dependent variable in our model. This variable has been measured with two items (α = 0.752) adapted from the “TPB Questionnaire Construction” guide (Ajzen, 2002). Table 2 indicates the items of the questionnaire.

### Analysis Method and Sample Description

IBM SPSS Statistics 26.0 and AMOS 23.0 were used to analyze the data. IBM SPSS Statistics 26.0 was used to calculate and describe the sample data, and AMOS 23.0 was used to analyze the structural equation.

We calculated the basic characteristics of the respondents by IBM SPSS STATISTICS 26.0, including gender, age, work years, family size, Know apps, etc. And Cronbach’s α was used to measure the reliability of the samples for potential constructs. AMOS23.0 was used to measure the structural equation model, and the maximum likelihood estimation method was used to analyze the measurement model and the structural model. Behavioral bias variables are added on the TPB model, which helps us to understand farmers’ mentality and better design communication strategies effectively (Mingolla et al., 2019). We used the mean to replace all the missing values. To measure the reliability of the questionnaire, we report Cronbach’s α. For testing the fitness of the structural equation model, we report X2/DF, GFI, RMSEA, IFI, TLI, and CFI as well.

## Results

### Descriptive Analysis

The respondents consisted of 219 aquaculture farmers, including 137 men and 82 women. The respondents’ age ranged from 17 to 67, with an average age of 37.68. The average working years of the respondents are 7.15 years, and 67.5% respondents know other similar platforms or software (see Table 1).

### Measurement Model

IBM SPSS Statistics 26.0 was used to measure the reliability of the questionnaire. Cronbach’s α of all variables is greater than 0.7 (see Table 2). In our model, default bias belief and bandwagon bias belief are manifest variables for better fitness so that Cronbach’s α could not be obtained. The final result showed that the data had good reliability (see Table 3). *p < 0.05; Besides, IBM SPSS 26.0 software was used to obtain Pearson correlation coefficient (Table 4); the result shows that the correlations were significant at the 0.01 level (two-tailed). It verifies that there are good correlations between variables.

**TABLE 3:** Parameter estimates and r-square.

Indicator	β	r-square
att_1	0.67	0.45
att_2	0.80	0.64
att_3	0.81	0.66
sn_1	0.80	0.64
sn_2	0.79	0.62
sn_3	0.57	0.33
pbc_1	0.78	0.60
pbc_2	0.76	0.58
pbc_3	0.84	0.71
ava_1	0.78	0.61
ava_2	0.74	0.55
los_1	0.78	0.61
los_2	0.75	0.57

*Default bias belief and bandwagon bias belief are manifest variables.*

**TABLE 4:** Pearson correlation coefficient.

Variables	ban	def	los	ava	ATT	SN	PBC	ADO
ban	1							
def	0.600**	1						
los	0.622**	0.665**	1					
ava	0.696**	0.629**	0.700**	1				
ATT	0.475**	0.479**	0.446**	0.452**	1			
SN	0.596**	0.570**	0.624**	0.608**	0.562**	1		
PBC	0.674**	0.638**	0.681**	0.682**	0.618**	0.680**	1	
ADO	0.695**	0.682**	0.687**	0.664**	0.634**	0.717**	0.797**	1

**The correlation was significant at the 0.01 level (two-tailed).

### Structural Model

**FIGURE 2:**
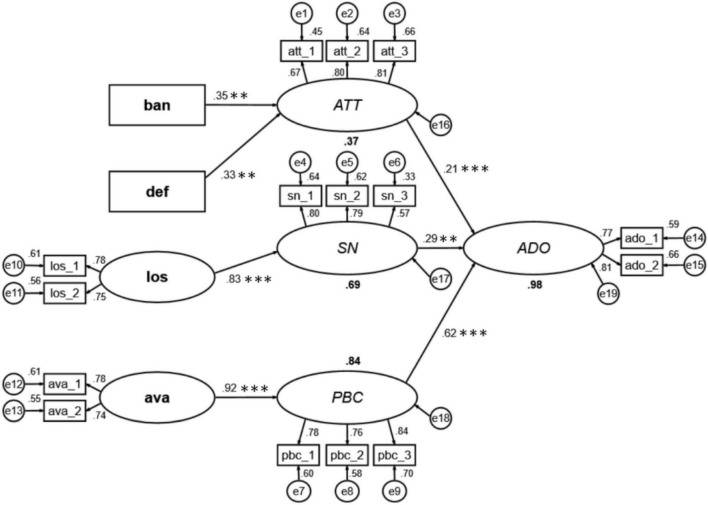
The structural equation model. The determinants of the TPB are indicated in font italic; the biased beliefs are indicated in font bold. ATT, attitude; SN, subject norms; PBC, perceived behavior control; ADO, farmers’ adoption intention; ban, bandwagon bias belief; def, default bias belief; los, loss aversion bias belief; ava, availability bias belief. We have only shown the important paths. ^**^p < 0.01 and ^***^p < 0.001.

Our structural equation model has a good fitness (see Table 5); x2/df = 2.268; GFI = 0.88; RMSEA = 0.76; IFI = 0.94; TLI = 0.924; CFI = 0.939. It is found that attitude, perceived behavioral control, and subjective norms have a positive effect on adoption intention, among which perceived behavioral control has the strongest positive effect on adoption intention, followed by subjective norms and attitude (see Figure 2).

**TABLE 5:** Fitness of the structural equation model.

Measure item	Level of acceptance fit	Fit statistics
x^2/df	<5 acceptable;<3 good	2.268
GFI	>0.8 acceptable;>0.9 good	0.880
RMSEA	<0.1 acceptable;<0.08 good	0.760
IFI	>0.9	0.940
TLI	>0.9	0.924
CFI	>0.9	0.939

In Table 6, biased beliefs have an indirect effect on adoption intention. Firstly, bandwagons bias belief has indirect effect on ADO through attitude (β = 0.076, *p* = 0.001). Secondly, default bias belief has indirect effect on ADO through attitude (β = 0.070, *p* = 0.001). Thirdly, loss bias belief has an indirect effect on ADO through subjective norms (β = 0.241, *p* = 0.035). Fourthly, availability bias belief has an indirect effect on ADO through perceived behavioral control (β = 0.573, *p* = 0.003).

**TABLE 6:** Standardized indirect effects of biased beliefs on behavioral intention.

Path	Estimate	SE	*p*-value
Bandwagons bias belief→ Attitude→ ADO	0.076	0.032	0.001
Default bias belief→ Attitude→ ADO	0.070	0.030	0.001
Loss aversion bias belief→ Subjective norms→ ADO	0.241	0.269	0.035
Availability bias belief→ PBC→ ADO	0.573	0.280	0.003

*PBC, perceived behavioral control; ADO, adoption intention.*

## Discussion

From private and public policies’ point of view, our results provide insights that can be used to understand farmers’ motivation to adopt the digital agricultural management system and develop strategies to stimulate the farmers’ adoption of the system by understanding a farmer’s mindset from behavioral theories and TPB theory. In this research, behavioral economics and the TPB model are utilized to predict farmers’ intention. The results show that biased belief can help to better explain why farmers have no enough motivations to adopt the digital agricultural management system, although it can benefit the entire production chain. Based on behavioral economics, this research examined how farmers’ belief toward the digital agricultural management system may be biased as a result of behavioral biases related to the perceived production losses and evident diseases (availability bias), perceived cost associated with the digital agricultural management system (loss aversion bias), custom to adopt their default management method (default bias) and the attitude of others toward the digital agricultural management system (bandwagon bias).

The results of this research portray that farmers attach great importance to the availability of the digital agricultural management system (availability bias). As demonstrated by the field survey data, more than 83.6% of the farmers have suffered a loss due to non-use or misuse of drugs. It is reasonable to submit that the management inefficiencies and unscientific aquaculture methods might cause the outbreak of disease and financial losses in the production chain. Once the digital system is considered as a reliable and scientific method to solve those problems, preventing future losses and raising economic profit and social benefits, farmers will be surer that they have more resources and opportunities to reduce the difficulty to manage, with less prospective barriers and more confidence to control (PBC). The fewer obstacles a person anticipates, the greater the perceived control over behavior (Vande Velde et al., 2015). This elucidates that framing recommendations in terms of losses (i.e., energy and money lost *via* the traditional management method) and the usefulness of this digital system may be more effective.

Furthermore, the results show that the intention to adopt positively is influenced by the loss aversion bias belief. The adoption intention of the digital agricultural management system is influenced more by financial costs (is it affordable?), physical risks (is it reliable?), social costs (is it friendly?), ecological risk (is it less polluting?), time costs (is it fast?), functional risks (does it suit me?), and even psychological costs (will I feel better?) instead of equivalent gains and benefits. The results revealed that farmers are likely to focus on the costs (i.e., time, effort, money) associated with the digital system, and how the digital system will assist them to prevent future losses and costs. Moreover, given that farmers have the aversion of losses, using simplifying heuristics to process information, they will pay more attention to other farmers’ costs and losses due to non-adoption of such a method, increasing subjective norms to have a higher intent to adopt this system. Thus, a statement such as “What you are doing currently is a couple of times less efficient than adopting the digital system” is likely to be more motivating than stating, “adopting the digital system is a couple of times more efficient than what you are currently doing.” In short, framing recommendations in terms of loss rather than gain may be more effective.

In addition, the findings of this research indicated that farmers only have a moderately bandwagon biased belief without a strongly belief that other farmers will actively adopt the digital management system. This may be because small-scale aquaculture production is predominant in the country, and most small-scale breeding farmers are very aged men without ability to use an intelligent device and digital system. Therefore, it is essential to strengthen user training and simplify operation to lower the threshold of the system. Nevertheless, this bandwagon bias belief does influence the farmers’ attitudes toward the digital system positively. Farmers will have a higher intent and more positive attitude to adopt the system when others are more proactive toward this system. The results highlight that it is more urging and motivating to frame using the digital system as both common and socially desirable, particularly advising farmers that others similar to them are using this digital system, or comparing a farmer’s social benefit and financial profit to that of farmers who do not adopt the digital system.

Besides, evidence from this study shows that the default biased beliefs seem to be the least strongly developed. The farmers in our sample did not strongly insist on their default traditional management method. It may because that more than 83.6% of the farmers in our field survey have already suffered a loss under their traditional breeding and the management method. It is expected that interventions focused on strengthening user training and simplifying operation to lower the threshold of the system and promoting the adoption of the digital system by framing using the digital system as both common and socially desirable.

The current investigation was the initial attempts for predicting farmers’ intention to adopt digital agricultural management system in a context of the TPB and behavioral economics applications, which aimed at combining the TPB and behavioral economics constructs and proposing an integrative model to address the limitation of TPB. Results of the survey revealed that attitude is being formed by beliefs related to the bandwagon bias and default bias, and subjective norms are being formed by beliefs related to the loss aversion bias, while PBC is being formed by beliefs related to the availability bias. These findings portray that biased beliefs do influence farmers’ rational thinking, enhanced the utility and predictive power of the TPB model for explaining and predicting farmers’ intention to use digital agricultural system. In general, the findings of the present research greatly improve our comprehension of the rational and irrational factors affecting farmers’ intentions to adopt the digital system besides providing a reference framework for the design and implementation of varied practical interventions by the relevant planners and policymakers to encourage the adoption of the digital agricultural management system among farmers.

There are also some limitations. First, our research only measured the behavioral intentions of the farmers and did not examine the actual behavior of the farmers. Although behavioral intention is a necessary condition and a strong explanation of actual behavior, it still cannot represent actual behavior. Therefore, a study based on actual behavior is suggested (Mingolla et al., 2019; Rezaei et al., 2019). Besides, this study only addressed the Chinese aquatic farmers in Guangdong province since Guangdong has the well-developed aquaculture; however, it cannot be claimed that the same results will be achieved in other areas. Therefore, it needs to be studied in other parts of China or the world with other farmers as well.

## Data Availability Statement

The raw data supporting the conclusions of this article will be made available by the authors, without undue reservation.

## Author Contributions

SS and XW wrote up the manuscript. SJ analyzed data. QG, YZ, and SW provided suggestions for this manuscript and revised the manuscript. All authors contributed to the article and approved the submitted version.

## Conflict of Interest

The authors declare that the research was conducted in the absence of any commercial or financial relationships that could be construed as a potential conflict of interest.

## Publisher’s Note

All claims expressed in this article are solely those of the authors and do not necessarily represent those of their affiliated organizations, or those of the publisher, the editors and the reviewers. Any product that may be evaluated in this article, or claim that may be made by its manufacturer, is not guaranteed or endorsed by the publisher.
